# Mass testing of healthcare workers for COVID-19—A single institution experience in Sabah, East Malaysia

**DOI:** 10.1371/journal.pone.0273326

**Published:** 2022-08-25

**Authors:** Wei Kong Wong, Audrey Shuk Lan Chong, Bing-Ling Kueh, Amirul Mohd Sallehuddin Bin Mannan, Muhammad Ubaidullah Arasy Bin Aziz, Zhi-Yiu Hiang-Weang Lim, Faulzan Bin Abdul Hamid, Marcus Netto, Bee Hwai Tan

**Affiliations:** 1 Traditional and Complimentary Medicine Unit, Sabah Women and Children Hospital, Ministry of Health, Kota Kinabalu, Sabah, Malaysia; 2 Clinical Research Centre (CRC), Sabah Women and Children Hospital, Ministry of Health, Kota Kinabalu, Sabah, Malaysia; 3 Quality Unit, Sabah Women and Children Hospital, Ministry of Health, Kota Kinabalu, Sabah, Malaysia; 4 Public Health Unit, Sabah Women and Children Hospital, Ministry of Health, Kota Kinabalu, Sabah, Malaysia; 5 Department of Pediatric Ophthalmology, Sabah Women and Children Hospital, Ministry of Health, Kota Kinabalu, Sabah, Malaysia; 6 Department of Nuclear Medicine, Sabah Women and Children Hospital, Ministry of Health, Kota Kinabalu, Sabah, Malaysia; 7 Department of Management, Sabah Women and Children Hospital, Ministry of Health, Kota Kinabalu, Sabah, Malaysia; The Chinese University of Hong Kong, HONG KONG

## Abstract

Approximately 1.29 million COVID-19 cases involving healthcare workers (HCWs) have been reported globally, leading to several hospitals conducting mass testing for early detection of infected HCWs. This study was conducted to report our experience and findings from the mass testing of HCWs from a public hospital in Sabah, Malaysia. The mass testing was conducted from 1^st^ March 2020 to 30^th^ June 2020, and involved self-reported data and laboratory results of 2089 HCWs. All HCWs who took at least two nasopharyngeal swabs for COVID-19 testing at two different time intervals during the study period were included. Throughout the mass testing period, various strategies such as practices of the new norm, daily temperature and symptom checking, wearing of appropriate personal protective equipment (PPE), identification of high-risk areas and travel declaration of staffs were within the hospital for prevention of COVID-19 transmission. We observed a small percentage of COVID-19 infected HCWs (n = 19, 0.91%) from the mass testing. Both symptomatic and asymptomatic COVID-19 HCWs were almost equal in number. A majority of those infected were nurses (n = 16, 0.77%) who had contact exposure to COVID-19 positive person or person under investigation (PUI) (n = 15, 0.72%). Four of the COVID-19 infected HCWs (n = 4/19, 21.05%) had no contact exposure. These HCWs were not identified through contact tracing. Fortunately, they were detected during the mass testing and were isolated promptly. In conclusion, mass testing of HCWs helped in early identification of COVID-19 infected HCWs not identified through contact tracing. Strategies such as stratified mass testing, strict compliance to new norm, appropriate PPE usage and identification of high-risk area were effective in the prevention of COVID-19 infection among HCWs.

## Introduction

The‌ ‌World‌ Health‌ ‌Organization‌ ‌(WHO)‌ ‌declared‌ ‌the coronavirus disease 19 (COVID-19‌) ‌as‌ ‌a‌ ‌global pandemic‌ ‌on‌ ‌11^th^‌ ‌March 2020 [1]. Following the worldwide declaration, a Movement‌ ‌Control‌ ‌Order‌ ‌(MCO)‌ was ‌enforced ‌by‌ ‌the‌ ‌federal‌ ‌government‌ ‌of ‌Malaysia on 18^th^‌ ‌March‌ ‌2020‌ ‌to prevent the spread of‌ ‌COVID-19 in Malaysia [2]‌‌. With the MCO in effect, the movement of persons and mass gatherings were prohibited. Only essential services related to water, electricity, food supplies, healthcare and defenses, were allowed to operate.

During the MCO period, treatment for ‌COVID-19‌ ‌and‌ ‌non‌ ‌COVID-19‌ ‌patients continued to be provided for by the healthcare workers (HCWs).‌ This put HCWs at a higher‌ ‌risk‌ ‌of‌ ‌being‌ infected by the disease as they were continuously exposed to COVID-19 patients.‌ ‌Approximately 1.29 million COVID-19 cases, or 8% of cases involving HCWs were reported globally as of January 2021 [3]. In Malaysia, a total of 1771 COVID-positive HCWs were recorded on 18 December 2020 [4], accounting for nearly 2% of Malaysia’s total cases.

Although‌ ‌strict‌ ‌preventive‌ ‌measures‌ ‌were‌ ‌imposed to reduce the risk of transmission, COVID-19‌ ‌infections‌ ‌among‌ ‌the‌ HCWs were ‌inevitable. Therefore, contact tracing was a key strategy in early detection of COVID-19 infection within the community [5], including among the HCWs. Other than contact tracing, HCWs were also reminded to report to the relevant party if presented with COVID-19 symptoms, to self-quarantine and take a nasopharyngeal swab test for reverse transcription polymerase chain reaction (RT-PCR) test for confirmation of the infection.

However, the concern for silent transmission of COVID-19 by asymptomatic person remained, as they comprised of 40% to 45% of all cases [6]. This was especially worrying in a healthcare setting where an outbreak could lead to severe consequences to the healthcare facility. ‌There‌ ‌were‌ ‌increasing‌ ‌calls‌ ‌for‌ ‌universal‌ ‌screening‌ ‌of‌ HCWs‌ ‌with‌ ‌COVID-19‌ ‌infections [7].‌ ‌Some studies‌ ‌suggested‌ ‌regular‌ ‌mass‌ ‌testing‌ ‌to identify‌ ‌and isolate‌‌‌ ‌asymptomatic‌ ‌COVID-19‌ ‌positive‌ ‌HCWs‌ ‌earlier,‌ ‌thus‌ ‌reducing‌ ‌nosocomial transmission‌ ‌to‌ susceptible‌ ‌patients‌ ‌and‌ ‌other‌ ‌HCWs, and‌ ‌stopping further‌ ‌spreading‌ ‌of COVID-19‌ ‌to‌ ‌the‌ community [8, 9].

The state of Sabah reported its first case of COVID-19 infection in a HCW on 26^th^ March 2020 (official statement form the Sabah Health Ministry). An epidemiological investigation revealed the source of infection from a cluster of COVID-19 cases involving HCWs of Sabah’s three major public hospitals. It became a concern to the Sabah Health Ministry then that if drastic measure was not taken, more COVID-19 clusters involving HCWs would emerge and threaten the operation of these healthcare facilities. Therefore, to detect and treat the infected HCWs early, and to curb the transmission of COVID-19 among them, a mass testing policy for the three major public hospitals was issued by the Sabah Health Ministry.

To date, there is a lack of published article about COVID-19 mass testing among HCWs especially in a hospital with limited resources. Hence, we would like to share our experience and findings in mass testing among HCWs in a public hospital and the strategies used to reduce the risk of COVID-19 transmission among HCWs.

## Methods

### Study design

This study involved the mass testing of COVID-19 among the HCWs cohort in a public hospital in Sabah during the period of 1^st^ March 2020 to 30^th^ June 2020. All‌ HCWs with‌ ‌at‌ ‌least‌ ‌2‌ ‌nasopharyngeal‌ swabs ‌taken for‌ RT-PCR test during the study period were included.

The data was extracted from the hospital’s database containing self-completed registration forms and swab results. The variables collected were age, gender, profession, workplace, epidemiology link, symptoms, use of PPE set, risk of exposure, swab dates, and swab results of all HCWs. Ethical approval was obtained from the Medical Research and Ethics Committee (MREC), Ministry of Health Malaysia before data collection. Individual consent was waived. [Reference number: KKM/NIHSEC/ P20-1350 (4)].

### Statistical analysis

Data were entered, cleaned, and analysed using SPSS 22.0 for Windows (SPSS Inc., 2013). Descriptive analysis was performed and reported by median and interquartile range for continuous variables; frequency and percentage for categorical variables.

Inferential analysis was done using multivariate logistic regression analysis, with the results of a positive COVID-19 RT-PCR test as the outcome variable. A pre-selection was performed using univariate logistic regression. Independent variables with *p*-value ≤0.25 were included in the full model. The full model was analysed using backward and forward stepwise Likelihood Ratio test; variables that were not significant were omitted from the full model. A *p*-value of ≤0.05 was considered statistically significant. A line chart was plotted using number of daily COVID-19 cases in Sabah, number of daily swabs and number of new cases of COVID-19 among HCWs from the public hospital from 1^st^ March 2020 to 30^th^ June 2020 for comparison of trend over time.

### The COVID-19 mass testing

The COVID-19 mass testing was facilitated by HCWs from the emergency department. Only trained doctors were allowed to take the swab specimens. Swab specimens were taken according to the Malaysian Ministry of Health guideline [10]. All facilitating HCWs were confirmed negative for COVID-19 before they were allowed to facilitate the mass testing. The swabbing was done in an open-air camp set outside the emergency department. All HCWs were required to self-complete an online registration form before they were registered for the swab test.

#### Exposed and non-exposed group

The HCWs were divided into two groups—exposed and non-exposed group. The exposed group were HCWs who had contact exposure with a COVID-19 infected person and were identified through contact tracing. Once identified, they were given the directive to immediately self-quarantine. Their first swab was taken on the 3rd day of contact exposure or immediately if the contact exposure had surpassed 3 days duration. They took the test on a walk-in basis. The non-exposed group were HCWs who had no contact exposure to COVID-19 infected person. This test was done on a scheduled basis. The appointment date was supervised by a senior colleague or a head of department to ensure that HCWs from the same department were tested in rotation. This was done to ensure that an adequate workforce was available at each department. All HCWs were required to self-quarantine after each swab. The turnover time for RT-PCR test was approximately 48 hours.

#### Test outcome and management

The HCWs were informed of the RT-PCR test results through a phone call. If the first RT-PCR test was negative, a second swab test would be arranged 48 hours after their first swab test. HCWs from the non-exposed group with two negative results and who were asymptomatic were allowed to return to their duties once they were notified by the Occupational Safety and Health Administration (OSHA) officer. However, if they remained symptomatic, a 14 days quarantine needed to be completed before they could resume work. For HCWs from the exposed-group with two negative swab tests, a 14 days quarantine must be completed before they resumed work regardless of whether they were symptomatic or asymptomatic. If the RT-PCR test was positive, the HCW would be contacted and admitted immediately to a COVID-19 treatment hospital until they tested negative or were no longer infectious.

#### PPE usage in the hospital

To avoid a shortage in the PPE supply, the level of PPE used was in accordance with the COVID-19 Management Guideline from the Ministry of Health [11]. A higher level of PPE usage (e.g. N95) was given to front-line HCWs who were involved in collecting a swab sample or managing suspected or confirmed COVID-19 person. If particulate respirators such as N95 was used, a fit test and user seal-check (fit check) was performed to ensure there was no leakage. For other HCWs, a surgical 3 ply mask and face shield or eye protection goggles were the minimal mandatory PPE for all HCWs in the hospital setting. As for patients, caretakers or visitors, a surgical 3 ply mask was the minimum mandatory PPE in the hospital setting.

#### Practice of new norm

Temperature scan and check-in using the MySejahtera mobile application was made compulsory for all individuals entering the hospital compound. No visitor was allowed into the COVID-19 wards. For non-COVID-19 ward, only one caretaker with confirmed negative test for COVID-19 was allowed to be in the ward with the patient.

All HCWs were reminded to practice physical distancing of at least 1-meter, proper hand hygiene and avoid crowded, closed or confined areas. Awareness was promoted rigorously through daily announcements via the public address system and infographic flyers. Hand sanitizers were allocated throughout the hospital including corridors for easy access. Smart paddle sanitizers were also introduced to reduce the risk of virus transmission through touch. Those caught violating the practices would be sternly warned and repeat offenders were compounded.

Physical meetings were converted to virtual meetings and dine-in at cafeteria was prohibited; only take-away was allowed. For non-clinical HCWs, they were allowed to work from home; as for clinical HCWs, they worked in rotation or bubbles to reduce the physical interaction between HCWs. All HCWs needed to notify their Head of Department and OSHA officer if they displayed any symptoms of fever with influenza-like illness (ILI) or Severe Acute Respiratory Infection (SARI).

#### Identification of high-risk areas

Hospital pantries were identified as high-risk area in view of direct exposure between HCWs during meal time. Furthermore, the number of pantries was limited, and the pantry area was small and poorly ventilated. Therefore, the pantry usage was limited to two persons at a time for a duration of ten minutes. Acrylic dividers were also placed on the table to act as barrier between the people dining at the same table. The ward counters were also identified as a high-risk area. These places were constantly monitored to prevent over-crowding.

#### Other measures for precaution

The hospital implemented a compulsory COVID-19 RT-PCR test for all new staff. They had to have two negative results before they were allowed to begin their service. Another additional measure taken was the introduction of a travel declaration form where all HCWs needed to declare all inter-district and inter-state travel to the OSHA officer. Once they returned from their travel, they would be screened and notified by the OSHA officer if they needed to be tested again before they resumed work.

## Results

### Response rate, demographic details with descriptive results

A total of 2167 (95.29%) out of 2274 HCWs from the hospital participated in the COVID-19 mass testing during the period 1^st^ March 2020 to 30^th^ June 2020. There were 2089 (91.86%) HCWs included in this study, 78 (3.43%) were excluded as they did not meet the inclusion criteria of having 2 nasopharyngeal swabs taken. A total of 4574 nasopharyngeal swabs were taken, with an average of 2 nasopharyngeal swabs taken from each HCW; only 192 HCWs had taken 3 or more nasopharyngeal swabs. Table 1 shows the demographic characteristics of the HCWs.

**Table 1:** Demographic characteristics of the HCWs.

Variable	*f*	COVID +ve	COVID -ve
	*n = 2089 (%)*	*n = 19 (*%)	*n = 2070* (%)
**1.Age group (year)**			
• **18–34**	1349 (64.58)	10 (0.48)	1339 (64.10)
• **35–50**	649 (31.07)	5 (0.24)	644 (30.83)
• **51–67**	91 (4.36)	4 (0.19)	87 (4.16)
• **Median (IQR)**	32 (IQR 28–37)*	34 (IQR 29–47)*	32 (IQR 28–37)*
**2.Symptom**			
• **Yes**	342 (16.37)	9 (0.43)	333 (15.94)
• **No**	1747 (83.63)	10 (0.48)	1737 (83.15)
**3.Gender**			
• **Female**	1483 (70.99)	19 (0.91)	1464 (70.08)
• **Male**	606 (29.01)	0	606 (29.01)
**4.Department**			
• **Clinical**	1581 (75.68)	18 (0.86)	1563 (74.82)
• **Non-Clinical**	508 (24.32)	1 (0.05)	507 (24.27)
**5.Workplace**			
• **COVID area**	634 (30.35)	11 (0.53)	623 (29.82)
• **Non-COVID area**	1316 (63.00)	8 (0.38)	1308 (62.61)
• **Missing**	139 (6.65)	0	139 (6.65)
**6.Profession**			
• **Doctor**	436 (20.87)	2 (0.10)	434 (20.78)
• **Nurse**	725 (34.71)	16 (0.77)	709 (33.94)
• **Allied health**	497 (23.79)	0	497 (23.79)
• **Ancillary staff**	324 (15.51)	1 (0.05)	323 (15.46)
• **Admin**	107 (5.12)	0	107 (5.12)
**7.Epidemiology link**			
• **No contact exposure**	1402 (67.11)	4 (0.19)	1398 (66.92)
• **First contact exposure**	566 (27.09)	14 (0.67)	552 (26.42)
• **Second contact exposure**	75 (3.59)	0	75 (3.59)
• **Contact with PUI**	27 (1.29)	1 (0.05)	26 (1.24)
• **Second degree contact**	12 (0.57)	0	12 (0.57)
• **Indirect contact**	7 (0.34)	0	7 (0.34)
**8.PPE**			
• **Yes**	954 (45.67)	10 (0.48)	944 (45.19)
• **No**	149 (7.13)	6 (0.29)	143 (6.85)
• **Breach**	12 (0.57)	0	12 (0.57)
• **Not relevant**	922 (44.14)	3 (0.14)	919 (43.99)
• **Unknown**	34 (1.63)	0	34 (1.63)
• **Missing**	18 (0.86)	0	18 (0.86)
**9.Risk of exposure**			
• **No identifiable risk**	1140 (54.57)	4 (0.19)	1136 (54.38)
• **High risk**	133 (6.37)	3 (0.14)	130 (6.22)
• **Medium risk**	440 (21.06)	8 (0.38)	432 (20.68)
• **Low risk**	102 (4.88)	4 (0.19)	98 (4.69)
• **Missing**	274 (13.12)	0	274 (13.12)

*data is skewed to the right

The median age of the HCWs was 32 years (IQR 28–37), with majority being female (n = 1483, 70.99%). More than half of the HCWs (n = 1349, 64.58%) were in the younger age group (18–34 years). The highest number of professions was contributed by the nurses (n = 725, 34.71%); followed by allied health staff (n = 497, 23.79%); doctors (n = 436, 20.87%); ancillary staffs (n = 324, 15.51%) and administration staffs (n = 107, 5.12%). Three-quarters were working in the clinical department (n = 1581, 75.68%) and almost one third were working in the COVID-19 treating area (n = 634, 30.35%) (information missing in n = 139, 6.65%). Less than one fifth of the HCWs were symptomatic (n = 342, 16.37%).

### HCWs with positive COVID-19 test

A total of 19 HCWs tested positive for COVID-19 (0.91%). Their median age was 34 years (IQR 29;47). All of them were female and most were nurses (n = 16, 0.77%). Nearly half of the infected HCWs were symptomatic (n = 9, 0.43%). The symptoms presented were cough (n = 8), fever (n = 5), sore throat (n = 3), runny nose (n = 2), and dyspnea (n = 1).

**Fig 1:**
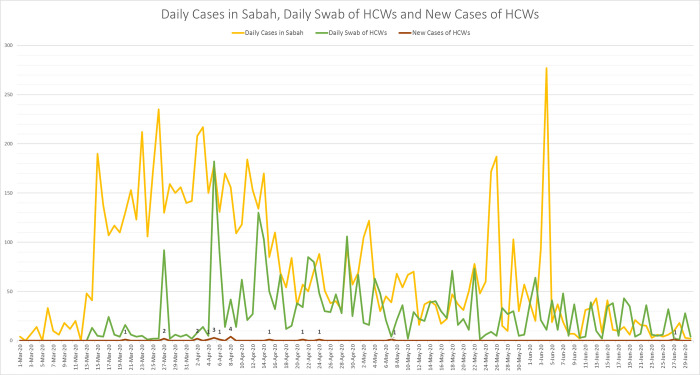
Daily cases in Sabah, daily swab of HCWs and new cases of HCWs. Daily cases of COVID-19 in Sabah, daily swab test for HCWs and new cases of COVID-19 infected HCWs recorded between 1 March 2020 and 30 June 2020; majority of HCWs were infected during the first wave of COVID-19 in Sabah.

Most of the COVID-19 infected HCWs were infected during the first wave of COVID-19 in Sabah (Fig 1). Fifty eight percent were working in the COVID-19 area (n = 11/19, 57.89%), and majority had contact exposure to COVID-19 positive person or PUI (n = 15/19, 78.95%). Twenty-one percent (n = 4/19, 21.05%) did not have a recent travel history or contact exposure with COVID-19 positive individual. HCWs with a medium risk of exposure recorded the highest number of COVID-19 positive cases (n = 8/19, 42.11%), and half of the HCWs (n = 10/19, 52.63%) wore adequate PPE at work.

Based on the epidemiological investigation, the source of infection for 8 HCWs were likely in-hospital transmission, 2 HCWs were likely community acquired while the remainder (n = 9) were unidentifiable source of infection.

### Multivariate analysis

Multivariate logistic regression analysis was done and shown in Table 2. Only three variables—age group, symptoms and risk of exposure—fit into the final model and were statistically significant. HCWs of the age group 51 years and above were about 8 times of odds of having COVID-19 compared to their younger peers (OR = 8.29, p = 0.001, CI 2.36–29.12). Symptomatic HCWs were almost 3 times of odds of having COVID-19 compared to asymptomatic HCWs (OR = 2.76, p = 0.043, CI 1.03–7.39). Lastly, HCWs with medium and low risk of exposure were about 4 times (OR = 4.51, p = 0.024, CI 1.22–16.59) and 9 times (OR = 9.41, p = 0.003, CI 2.16–40.92). respectively, of odds of having COVID-19 compared to those without identifiable risk of exposure.

**Table 2:** Univariate and multivariate logistic regression analysis showing odds ratio of factors associated with COVID-19 infection among the HCWs.

Variable	Univariate logistic regression analysis			Multivariate logistic regression analysis					
				Model 1			Model 2		
	Crude OR	(95% CI)	p-value	Adjusted OR	(95% CI)	p-value	Adjusted OR	(95% CI)	p-value
**1.Age (year)**									
• 51 and above	6.16^b^	(1.89–20.03)	0.944	7.77^a^	(1.92–31.44)	0.014	8.29	(2.36–29.12)	0.001
• 35–50	1.04	(0.35–3.05)	0.003	1.23	(0.41–3.72)	0.718	1.15	(0.39–3.42)	0.805
• 18–34 (RC)	1.00			1.00			1.00		
**2.Symptoms**									
• Yes	4.70^c^	(1.89;11.64)	0.001	2.63	(0.93–7.42)	0.068	2.76	(1.03–7.39)	0.043
• No (RC)	1.00			1.00			1.00		
**3.Gender**									
• Female	2.10 x 10 ^7^	(0.00-NA)	0.992	-	-	-	-	-	-
• Male (RC)	1.00								
**4.Department**									
• Clinical	5.84	(0.78–43.85)	0.086	3.91	(0.46–33.19)	0.211	-	-	-
• Non-clinical (RC)	1.00			1.00					
**5.Workplace**									
• COVID area	2.89^a^	(1.15–7.21)	0.023	1.84	(0.68–5.03)	0.233	-	-	-
• Non COVID area (RC)	1.00			1.00					
**6.Profession**									
• Doctor	7.44 x 10 ^6^	(0.00-NA)	0.997						
• Nurse	3.65 x 10 ^7^	(0.00-NA)	0.996	-	-	-	-	-	-
• Allied health	1.00	(0.00-NA)	1.000						
• Ancillary staff	5.00 x 10 ^6^	(0.00-NA)	0.997						
• Admin (RC)	1.00								
**7.Epidemiology link**									
• First contact exposure	8.86^c^	(2.91–27.05)	<0.001	8.78	(0.29–264.86)	0.212			
• Second contact exposure	0.00	(0.00-NA)	0.997	0.00	(0.00-NA)	0.997	-	-	-
• Contact with PUI	13.44^a^	(1.45–124.44)	0.022	20.26	(0.36–1130.03)	0.143			
• Second degree contact	0.00	(0.00-NA)	0.999	0.00	(0.00-NA)	0.999			
• Indirect contact	0.00	(0.00-NA)	0.999	0.00	(0.00-NA)	0.999			
• No contact exposure (RC)	1.00			1.00					
**8.PPE**									
• No	3.96^b^	(1.42–11.06)	0.009	8.62^b^	(1.61–46.22)	0.012			
• Breach	0.00	(0.00-NA)	0.999	0.00	(0.00-NA)	0.999	-	-	-
• Not relevant	0.31	(0.09–1.12)	0.074	2.29	(0.26–20.58)	0.459			
• Unknown	0.00	(0.00-NA)	0.998	0.00	(0.00-NA)	0.999			
• Yes (RC)	1.00			1.00					
**9.Risk of exposure**									
• High risk	6.55^a^	(1.45;29.61)	0.015	0.10	(<0.01–4.88)	0.246	4.06	(0.85–19.48)	0.080
• Medium risk	5.26^b^	(1.58;17.56)	0.007	0.70	(0.02–23.39)	0.844	4.51	(1.22–16.59)	0.024
• Low risk	11.59^c^	(2.86;47.06)	0.001	0.68	(0.02–24.52)	0.832	9.41	(2.16–40.92)	0.003
• No identifiable risk (RC)	1.00			1.00			1.00		

### Mortality

During the mass testing period, 1 HCW passed away due to cancer, with COVID-19 suspected as an indirect cause. The HCW tested positive through 4 antigen-based rapid test kits (RTK-Ag), but tested negative in RT-PCR tests for nasopharyngeal swabs, tracheal aspirate and tracheal swab.

The source of infection was unable to be identified through an epidemiological investigation. However, the HCW had been on medical leave of absence since before the COVID-19 pandemic and was admitted to the ward only during cancer treatment.

## Discussion

The mass testing in our public hospital detected only a small percentage of COVID-19 infected HCWs (n = 19, 0.91%). This percentage is similar to a study done by Oster et al [12]. Majority of the infected HCWs were detected during the initial wave of COVID-19 in Sabah. Subsequently, only two HCWs were detected to have COVID-19 infection over a span of two months (May- June 2020). This can be contributed to the MCO that was implemented in Malaysia on 18^th^ March 2020. Vincenzo et al stated that lockdowns are effective in limiting the number of new COVID-19 cases in countries that implemented it, and this benefit could extend beyond 20 days [13].

Most of the infected HCWs were nurses (n = 16/19, 84.21%) and had contact exposure to COVID-19 infected person or PUI (n = 15/19, 78.95%). This is likely due to the fact that the first infected HCW detected was also a nurse. However, four HCWs (n = 4/19, 21.05%) had no evidence of contact exposure to COVID-19 infected person or PUI. Therefore, the mass testing helped in early identification and isolation of HCWs who were not identified through contact tracing. The prompt identification helped in lowering cross-transmission between HCWs, reassuring the families of HCWs and patients, and ensuring the optimal functioning of the hospital during this pandemic [14].

In this study, there was a higher prevalence of COVID-19 positivity from the HCWs working in the clinical department (n = 18/19, 94.74%) and the COVID-19 area (n = 11/19, 57.89%). This is in contrast to a similar study by Diana et al. who observed a higher prevalence among HCWs from the non-clinical department and non-COVID-19 areas [15]. The difference could be due to many factors. One difference observed is the compliance in the practice of new norm and precaution measures taken especially in the high-risk areas such as the pantry and ward counters. Kaur et al shared that informal interactions such as having meal together, not maintaining physical distance, and inconsistent use of mask during routine work were common reasons for high-risk exposure [16]. Whereas, our hospital implemented precaution measures for high-risk area such as the pantry and ward counter usage from the start of the COVID-19 pandemic by limiting the number of staff and practiced constant monitoring. Furthermore, our HCWs were constantly reminded to practice the new norm inside and outside the hospital. The travel declaration form was also made compulsory for those travelling. These strategies could have contributed in maintaining a lower number of infections among the HCWs.

One mortality was reported among the COVID-19 infected HCWs. The cause of death was due to cancer, with COVID-19 suspected as an indirect cause. Although the RTK-Ag tests were consistently positive, but the post-mortem RT-PCR test results were negative. A systemic review showed false negative RT-PCR tests ranged from 2% to 29%. The viral load for COVID-19 could have been below the detectable level after the death of this HCW [17].

Lastly, the prevalence of symptomatic and asymptomatic COVID-19 infected HCWs were equal, but symptomatic HCWs are almost 3 times of odds of having COVID-19 compared to asymptomatic HCWs. Also, HCWs at age group 51–67 years are about 8 times of odds of having COVID-19 compared to their younger peers. These results highlight the importance of prioritizing screening among symptomatic HCWs and those from an older age group as they are at higher odds of getting infected by the disease. However, this does not mean that the screening of asymptomatic HCWs can be omitted, as they too can transmit the virus if not detected and isolated early [7]. We also reported higher odds of COVID-19 infection among HCWs with low to high risk of exposure to COVID-19 compared to HCWs without any identifiable risk. However, this could be a result of having a small number of COVID-19 positive HCWs in our study and warrants further study.

## Conclusions

Strategies such as stratified mass testing, strict compliance to the new norm, appropriate PPE usage and identification of high-risk area have been crucial to maintaining a lower number of infections among HCWs. We aim to continue implementing these strategies in accordance to the World Health Organization guidelines.

## Limitations and recommendation

The data was collected from a self-completed form, so they may have been recall bias. Symptoms such as anosmia were not included in the initial registration form, so it was not included in our study. It was not compulsory to answer all questions, resulting in 3 variables (workplace, PPE and risk of exposure) with missing data. The study was conducted in a single institution; hence the data is not representative of other institutions. Future study should include cost-effectiveness evaluation to determine whether the cost can be considered reasonable for the clinical gain.

## Supporting information

S1 File Data collection of all healthcare worker from 1^st^ March 2020 to 30^th^ June 2020. Sheet 1: Data of all healthcare workers included in data analysis; Sheet 2: Data of all COVID-19 positive healthcare workers; Coding: Label for codes used.
(XLSX)

S2 File Data collection of all healthcare worker from 1^st^ March 2020 to 30^th^ June 2020 (SPSS). (SAV)
